# Confluence of crises: COVID-19, "gassings", blood draws and the continued importance of community engagement in Zambia

**DOI:** 10.34172/hpp.2022.09

**Published:** 2022-05-29

**Authors:** Sara H. Olsen, Esther J. Roh, Tandwa Syakayuwa, Mumbi Chola, Chinedu Agbakwuru, Kristen A. Stafford, Kirsten Stoebenau, Kumbutso Dzekedzeke, Manhattan Charurat

**Affiliations:** ^1^Department of Behavioral and Community Health, University of Maryland School of Public Health, Maryland, USA; ^2^Center for International Health, Education, and Biosecurity, Zambia; ^3^Center for International Health, Education, and Biosecurity, University of Maryland School of Medicine, Maryland, USA; ^4^Institute of Human Virology, University of Maryland School of Medicine, Maryland, USA

**Keywords:** Blood, COVID-19, Community-based participatory research, HIV

## Abstract

**Background:** Nationally representative, household-based, health-related surveys are an invaluable source of health information, but face implementation challenges. In sub-Saharan Africa, these challenges are exacerbated when surveys include the collection of biological specimens. In this study, we describe the potential implementation challenges identified during field practice leading up to the 2020 Zambia Population-based HIV Impact Assessment (ZAMPHIA) survey, and explore the role of two crises on community mistrust of, and apprehension to, participate in the survey.

**Methods:** Using focus group methodology to better understand the influence of crises on ZAMPHIA participation, we conducted 12 focus group discussions (FGDs) in five districts across two provinces. FGDs were conducted with three purposively sampled study groups: recognized household heads, community leaders, and young adults aged 18-24 years. We used reflexive thematic analysis to develop themes from across the FGDs.

**Results:** We identified two key themes: the ever-present threat a stranger posed to the community is enhanced by crises, and endorsement of community awareness through sensitization can mitigate outsider challenges in medical research.

**Conclusion:** We argue that these crises emphasized underlying mistrust that can only be addressed with substantial investment in community engagement efforts to build trust and partnership in medical research endeavors. Our findings underline the importance of prioritizing community engagement through substantial investment in varied and extensive approaches to sensitization to facilitate community engagement toward community acceptance of ZAMPHIA and similar studies.

## Introduction

Nationally representative, household-based, health-related surveys are an invaluable source of health information in sub-Saharan Africa. One of the more recent of these large-scale, multi-country survey efforts is the Population-based HIV Impact Assessment project. These surveys are funded by U.S. government agencies and conducted in partnership with Ministries of Health in countries with high HIV (human immunodeficiency virus) prevalence. The results of the survey offer substantially more detailed insight on the state of the HIV epidemic than other nationally representative household-based health-related surveys in the region (e.g., Demographic and Health Surveys), which were only designed to estimate HIV prevalence.^1^ They collect data to address the needs for HIV testing, treatment, and treatment adherence targets that affect how HIV-related programs are resourced and implemented.

The surveys involve demographic and behavioral data and blood collection from households that have been selected through a two-stage sampling design. The survey aims to enroll all selected households, and eligible household members from within, by obtaining consent for interview and blood draw. The validity of the study findings is largely based on the response rates at the household and individual level, making community and individual acceptance of the survey procedures paramount to its success.

Given the importance of this survey for program evaluation and resource allocation, it is essential that response rates are high and unbiased, and survey administration runs smoothly. Yet, as a household-based survey that includes a blood draw, and given that HIV testing is itself laden with stigma, Population HIV Impact Assessment surveys face significant implementation challenges. In particular, studies from contexts across sub-Saharan Africa have documented community concerns associated with blood collection that occurs outside of a medical facility. Particularly relevant in contexts where there is widespread belief that blood carries power and holds spiritual significance, concerns with blood draw have included the quantity of blood, the intended use of the blood, and the perceived effects of the blood draw on the individual body.^2-5^ Concerns with blood draw in household-based surveys have been reported in Zambia, in particular.^6,7^

Some social scientists have argued that community concerns with medical research projects involving blood draw often represent underlying concerns of power, position, and trust.^8^

In this paper we address implementation challenges for a specific HIV impact assessment, the 2020 Zambia Population-based HIV Impact Assessment (ZAMPHIA), which was set to begin data collection in early 2020. Two crises occurred in Zambia concurrent with preparations for the ZAMPHIA survey and held implications for data collection: the COVID-19 pandemic, and immediately prior to that, a more local gassing crisis described below. As we detail in this paper, these crises served to exacerbate existing concerns for HIV studies that include a community-based blood draw.

## Global and national crises and the ZAMPHIA survey

The Zambian gassing crisis began with scattered reports of household burglaries in late 2019. Allegedly, thieves would release a gas into the home that rendered residents unconscious and then commit the theft – hence the local term “gassings.” Reports of gassings became widespread and pervasive in news and social media.^9,10^ As fear engulfed many communities, gassings became increasingly described as accounts of blood theft, sometimes en masse.

Simultaneous to the reported gassing incidents, ZAMPHIA began mass media and other campaigns to provide information about the upcoming survey, followed closely by a pilot test in selected communities during February, 2020. Some pilot test field teams, suspected of being gassers, were apprehended, threatened, or detained. Further, a social media analysis of the term “ZAMPHIA” indicated that it was becoming associated with gassing on social media posts in the country.

The COVID-19 pandemic reached Zambia in April, 2020. In order to ensure the safety of both enumerators and community members, and given the state of the science at the time, ZAMPHIA enumerators were expected to wear full personal protective equipment (PPE) during data collection activities. This expectation added another layer of complexity, however, as it was alleged that gassers wore masks and similar gear.

This study seeks to understand the dual gassing and COVID-19 crises’ influence on community level trust as it relates to: (1) study enumerators as community outsiders; and (2) the implications of community trust on future community partnerships in public health research and practice. We suggest that underlying the concerns with blood draw across communities was deep-seated mistrust, and we argue that this mistrust was amplified, but not generated, by these crises. Our findings highlight the importance of active community engagement in the entire research process to minimize community fears, and inform future work in Zambia and similar contexts.

## Materials and Methods

This study employed thematic analysis to explore, analyze, and report patterns among the focus group discussions (FGDs).^11,12^ We used a realist approach to report the participants’ experiences and meaning made of those experiences.

### Research setting and recruitment

The study took place in two provinces and five districts of Zambia** (**Table 1). These included the Lusaka Province—which encompasses a district and national capital city of the same name—and in the contiguous Southern Province. Study sites were purposively selected to capture both rural and urban community perspectives in contexts where there were both (1) previously reported incidents of gassing, and (2) exposure to ZAMPHIA messaging.

**Table 1:** Focus Group recruitment distribution

**Province**	**District**	**Residence**	**Enumeration area**	**Heads of household**	**Community leaders**	**Young adults**
Southern	Choma	Urban	Railways	×		×
	Pemba	Rural	Jembo		×	
	Mazabuka	Peri-urban	Ndeke-Zambia			×
	Choma	Urban	Shampande	×	×	
Lusaka	Lusaka	Urban	Chainama	×		×
			Chainda		×	
	Chongwe	Rural	Powanga			×
			Mutamino	×	×	

With respect to participant inclusion and exclusion criteria, households that had been sampled for the ZAMPHIA pilot were excluded from participation in this FGD research. Within each province, the team purposively sampled participants representing three groups: household heads, community leaders (e.g., head teachers, councilors, Ward Development Committee chairpersons, headmen, Indunas, religious leaders) and young adults aged 18-24 years who reside with parents or guardians. These groups represented different components of the community whose engagement and buy-in were deemed necessary for ZAMPHIA’s success. Inclusion criteria included: age (18 years and older), residence in the community, and primary language (English, Bemba, Nyanja, or Tonga).

Two community mobilizers per enumeration area were recruited and trained to conduct participant recruitment based on study group and eligibility criteria. Individuals that met the inclusion criteria were purposively selected to participate in the FGDs. Individuals with cognitive impairment and those who had participated in the pilot field study were excluded.

Informed consent was signed by all participants and obtained in English, Bemba, Nyanja or Tonga depending on participant preference. The consent process involved describing the study and its purpose, liberty to withdraw, confidentiality and reimbursement for travel to the study site. Consent was reviewed word-for-word with each participant.

### Data collection

Twelve FGDs were conducted in four enumeration areas per province in July-August, 2020** (**Table 1). Two research teams (one in each province) of six staff collected the data. All staff were tested for COVID-19 before they went in the field, and COVID-19 safety measures were followed throughout recruitment, data collection and analysis. Each FGD was facilitated by two experienced facilitators (one moderator and one note taker). Semi-structured FGD guides were used. The focus group questions were designed to gauge ZAMPHIA awareness and understanding as well as acceptability of enumerator COVID-19 precautions during future data collection. The guides included open-ended questions covering topics, such as knowledge of ZAMPHIA, ZAMPHIA survey participation acceptance, reflections on gassing incidents and blood draws, knowledge about COVID-19, and perceptions about community acceptability of PPE. Examples of questions included: *What are your concerns for your community testing for HIV? What are your concerns for your community about giving blood for further testing? Did you ever hear about the gassing incidents? Share your experience.* All FGDs were audio-recorded, transcribed verbatim, and then translated to English for coding. Transcriptions and translations were conducted by the research team that collected the data and third-party transcribers. Both sets were reviewed by the team leads ahead of data analysis. English transcripts were loaded into a software package ATLAS.ti version 9.0 (ATLAS.ti Scientific Software Development GmbH, Berlin, Germany) for the data analysis.

### Data analysis

**Figure 1:**
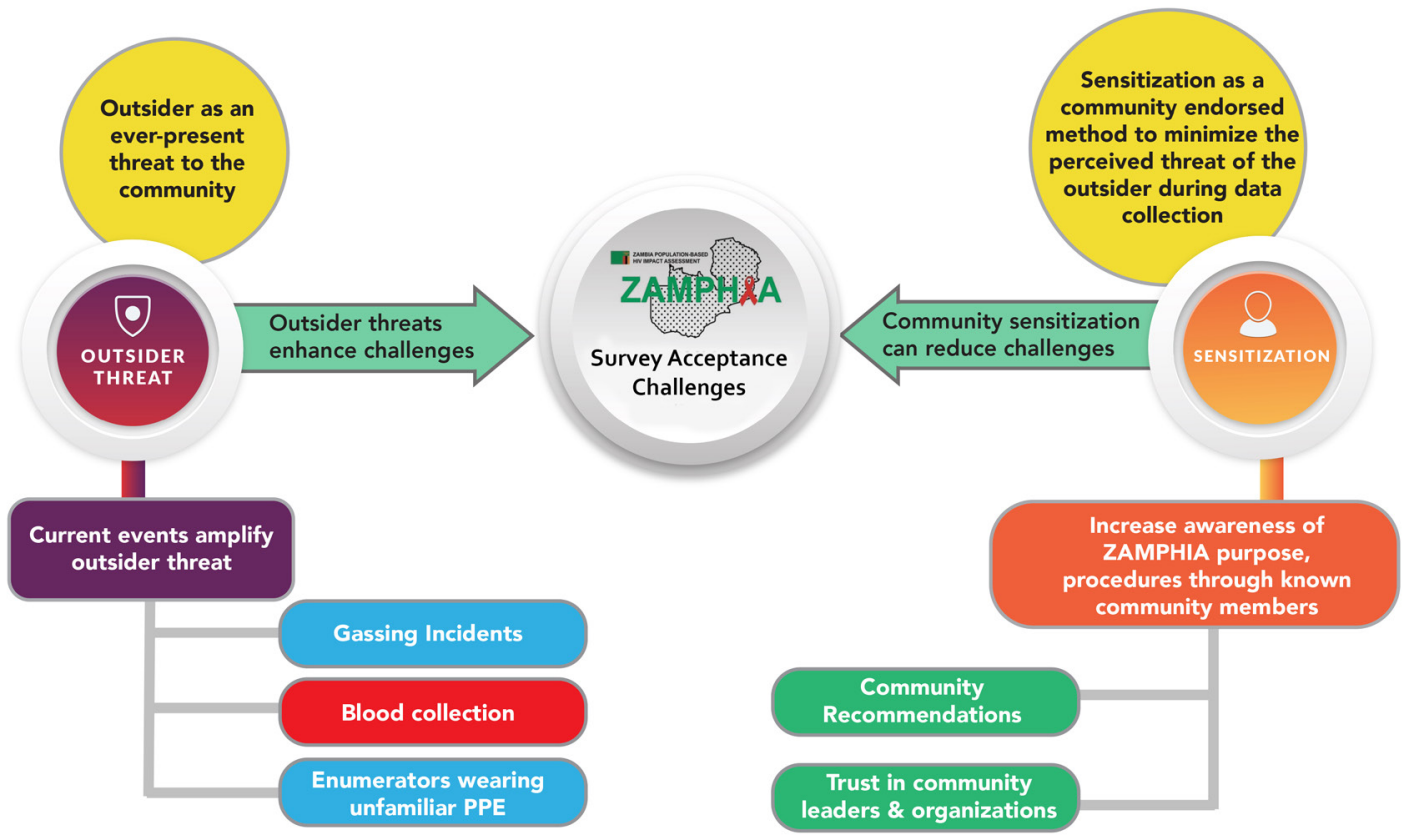
Thematic Concept Map resulting from FGD analysis

FGD data were coded by two researchers. Data analysis followed a five-step thematic analysis process^11^: (1) Researchers separately read the English translation of transcriptions several times to familiarize themselves with the data; (2) the research team generated initial deductive codes based on the FGD guide and two researchers analyzed the same FGD, line-by-line. Following coding of an additional subset of transcripts, the initial codebook was revised and refined with inductive codes (Table 2). (3) Once both deductive and inductive codes were applied to all FGDs, the codes were then compared and grouped by candidate themes^11^; (4) the relationships between candidate themes were compared and interpreted, forming a thematic map (Figure 1). (5) Finally, selected themes and subordinate extracts were compared for data accuracy and internal consistency and sufficient data to support the central concepts represented by the themes.^12^ In the final step, the themes and subthemes were compared with the source data and reviewed by the data collection team in Zambia to verify they accurately reflected the participant perspective.

**Table 2:** Sample Codes and their definitions from the final codebook

**Sample code**	**Sample code definition**	**Resultant major theme**
beliefs about PPE	Community’s beliefs and opinions on PPE use among the community members, how it influences younger generations (children and youth under 18) and who influences them	The outsider as an ever-present threat to the community
Gassing threat	Community’s experience in any gassing incidents	
Blood collection positive	How blood collection would help community on identifying HIV and testing HIV	
Blood collection negative	Negative beliefs or concerns associated blood collection	
ZAMPHIA positive	Comments in which the survey is perceived as beneficial to individuals or communities	Sensitization as a community endorsed method to reduce perceived outsider threat
ZAMPHIA negative	Reasons why the survey may be perceived as stigmatizing, untrustworthy, or otherwise unwelcome in the community	
Survey participation positive	Reasons why participants or community want to participate in the survey	
Survey participation negative	Reasons why participants or community not want to participate in the survey	
ZAMPHIA communication	Mechanisms by which the participants gained knowledge of the survey, formal and informal	

Note: The third column indicates the Themes that were developed using, in part, the sample codes provided.

### Qualitative rigor

Trustworthiness and credibility were ensured through several measures. Codes were independently developed by the analysis team and internal verification occurred by comparing code definitions. Analysts also codes the same FGD transcript to ensure codes were being applied to the data in the same manner. Multiple meetings were held with the field team throughout the process of codebook and theme development. As part of ensuring the analysts were capturing the sentiments of participants, a preliminary thematic map with illustrative data extracts were shared with the field teams that were present during FGDs. Key stakeholders provided feedback of preliminary and final results.

## Results

Overall, two primary and interconnected themes were identified in the data. These themes, corresponding sub-themes, and relationships between them as identified through the analysis, are detailed in Figure 1, which summarizes the results from this study. The main themes are introduced here and then presented in detail below. The first main theme was the ever-present threat a stranger or person deemed as an “outsider” posed to the community and to individuals within the community. This theme emerged through participant discussions concerning community concerns associated with blood draw for HIV tests and ZAMPHIA enumerator use of PPE for prevention of COVID-19 transmission. Participants expressed concerns with outsiders through reflections on recent crises including gassing attacks and the COVID-19 pandemic, which also intersected with underlying uncertainties surrounding the motivation for blood draw, and its intended use. The discussion and concerns about ZAMPHIA enumerators’ reception in communities was amplified as a result of it being interpreted through the lens of these recent crises. The second theme was the endorsement of improving community awareness of ZAMPHIA through sensitization as a way to reduce challenges to ZAMPHIA data collection. It is important to note that while participants across most sites and study groups shared some awareness of the ZAMPHIA study and familiarity with the ZAMPHIA name through mass media or local health workers, few had full knowledge of its purpose or how data were collected. Most participants suggested that increasing communities’ awareness of ZAMPHIA through community education and use of known community leaders and organizations would reduce fear and anxiety amongst community members.

### Theme 1: The outsider as an ever-present threat to the community

Overall, throughout the FGDs, participants expressed fear, concern, and mistrust of those identified as outsiders, or whose belongingness, and therefore trustworthiness, could not be readily ascertained. Though precautions associated with the COVID-19 pandemic and events related to gassing attacks provided new ways to conceptualize the outsider, the threat was the not knowing – not knowing identity, intent, motives. During the FGDs, the outsider threat was captured under three subthemes described below: the outsider as related to (1) the collection of blood for HIV testing as part of the ZAMPHIA study; (2) the recent gassing attacks throughout the country; and (3) the presentation of enumerators in PPE.

#### Blood collection and potential use creates worry

Despite some acknowledgement of the benefits to knowing one’s status that would result from access to HIV testing, the general attitude toward blood collection was one of trepidation. Blood collection was tied to mistrust of the outsider in two ways. First, FGD participants were worried about ulterior motives to the collection in which the blood would be used for ritualistic purposes. Second, FGD participants felt communications about blood collection processes were unreliable.

In each FGD, participants talked about their fear or their community’s fear that blood samples would be used for rituals, describing the collecting of blood as “linked to Satanism.” Participants expressed fear that the blood was being collected to benefit politicians, for monetary gain, and to hurt others. Ritual murders were tied to seasonal increases in crime and social media cautioned against participating in blood collection studies. A community leader in Lusaka, talking about the people in his community, described a deeply entrenched belief about the use of blood beyond medical purposes.

“*Their thinking could be that maybe these are Satanists who could be using blood for business or using it to cause harm…They may doubt where information would be taken and what would be done with it. Like I have already mentioned, we are approaching elections…Truly many will have misconceptions, especially on information and blood collection, thinking that maybe they want to sell my blood or do with it many other bad things.*”

The second concern centered on the HIV tests that would be conducted using the blood that was collected, and how results would be disseminated. Previous HIV test results had not been shared. FGD participants expressed frustration with the poor communication about the timing of the release of test results, how they would be delivered, and the processes in place to maintain confidentiality. Generally, FGD participants understood the importance of individual awareness of HIV status, as well as the ability for the government to track the spread or containment of the virus. Community leaders in both Lusaka and Southern Provinces stated they had not yet seen the results from the 2016 study. This lack of transparency and communication resulted in community members’ reluctance to welcome outside enumerators back. In addition, some participants expressed that they were never informed of their individual HIV test results from blood collected in 2016.

“*Now take for instance of what happened in the past 2016 the results are not yet availed and somehow there was a problem somehow maybe results were given somewhere else so that is the situation you have explained. All such kind of things are bringing a negative impact in the end you will say these people seem to be hostile you know*.” (Southern Province, Urban Community Leader).

The experience of not receiving HIV test results after the 2016 data collection heightened concerns that blood samples had been used in rituals. “*If results are not returned, people will begin to get worried and question where their blood samples were taken”* (Lusaka, Urban Community Leader). Blood needed for medical testing was understood and accepted; however, carrying blood away from the home created cause for worry. Participants explained that HIV testing in a clinic was conducted in a visible phlebotomy lab. They knew where their blood was going – it was “protected.” Blood collected at a person’s home would be handled by individuals not known to the community and transported elsewhere. This vague and unfamiliar process meant, to many participants, an opportunity for the blood to be used in ways other than promised:

“*Concern, suspicion, just like everyone else has stated, there are issues pertaining to Satanism so what people do not know well is, is it true the blood is going to the hospital or where is it going? So that is the main concern which people have - suspicion.*” (Lusaka, Rural Community Leader).

#### Gassing incidents increased community vigilance against outsiders

The gassing incident in 2019 through 2020 occurred simultaneous to the ZAMPHIA pilot study, and the survey team was concerned that gassing might be conflated in participants’ minds with the presence of ZAMPHIA enumerators. Most FGD participants had not experienced a direct gassing attack, but several had responded to nearby reported gassing incidents in an effort to protect their neighbors, or had spoken to neighbors who had been gassed. Additionally, participants reported reading about incidents on social media or hearing about events third-hand. For most participants, the gassing incidents were understood as a mechanism for blood theft and stoked fears of outsiders and their intentions. Specifically, many FGD participants believed the underlying purpose of gassing was to collect blood for ritualistic purposes, often in very large quantities.

“*This issue of gassing, me, I was just praying that the gassers will not come to my home because it was [scary] what they were doing when you are gassed and faint, they drain your blood. So, this blood they were taking it somewhere, I was told that they needed drums of blood.*” (Southern Province, Urban Community Leader).

In some cases, rumors about the amount of blood being taken from unwilling recipients were joined by mythological assertions, such as that gassers could shape-shift.

Because those committing the gassing attacks had not been identified, communities became more wary of anyone they did not know entering their compound. FGD participants described how the gassing incidents disrupted their daily routines, sleep, and caused them to live with constant fear. A young adult in Southern Province explained if they saw a “*new face...people will start saying that maybe these are the people associated with gassing, so it was quite very difficult for you as a person staying in that compound to welcome or help someone who does not stay in the community*.” Communities were living in fear. Stories of people who had been gassed in their own home and again when trying to seek medical help gave the feeling of no safe place: “ *people were just using their own source of means to survive*” (Southern Province, Urban Community Leader).

To shield against these unknown or outsider gassers, communities turned inward for protection, enacting hypervigilant countermeasures. Curfews were implemented or businesses would voluntarily close early. Some families and compounds set up patrols throughout the night and stayed together in one group to prevent being caught by surprise.

“*Yes, in our area gassing incidences were there…they were gassing people, this would scare people and they would go and spend nights in one group in the community, they feared sleeping separately, and ran away from their own homes, that is what transpired*” (Lusaka, Rural Community Leader).

One FGD participant explained that trusting someone takes a long time and gassing incidents reduced readiness to trust someone from outside the compound. ZAMPHIA began the 2020 pilot study as gassing incidents were becoming more of a perceived threat. Enumerators, as community outsiders, were mistaken in some places for gassers, frightening potential participants.

#### Full PPE hides identity of enumerator

During the FGDs, participants were shown images of enumerators in various levels of PPE ranging from a cloth face covering to an outfit—full PPE—with a surgical mask, face shield, goggles, disposable lab coat, and medical gloves. Participants discussed PPE as both an important precaution in the current COVID-19 pandemic but also a reason for suspicion. They were concerned about the message a person dressed in full PPE entering their homes would send to neighbors about their disease status. They also expressed concern over the fact that PPE concealed some or all of someone’s face, making enumerator identities uncertain.

Participants in all FGDs said they would readily accept an enumerator wearing only a face covering (face mask) because they were used to the practice as a result of local and national COVID-19 precautions. One participant viewed face cover wearing as a respectful measure, showing the enumerators wanted to protect the households as much as themselves. Another felt the face mask indicated the enumerator’s intent to provide support: “*I would be very humbled because in the sense that person is coming to help me. And the situation has come forth. Therefore, I need to take him in and welcome him with all the safety hospitality measures*” (Southern Province, Urban Head of Household).

However, when shown pictures of potential enumerators in full PPE (plasticized lab coat, face shield, N95 mask, protective goggles, gloves), there was immediate aversion. Full PPE, as pictured, was not familiar to FGD participants. They associated the lab coat with hospital and clinic professionals, and the full outfit was described as scary, ghost-like, surprising, and unfriendly. Full PPE, unlike a face covering, was perceived as protection from a known contagion within the home. FGD participants said they would turn away enumerators dressed in full PPE; fearful the full PPE indicated a diagnosis. FGD participants did not want neighbors to assume their home had COVID-19 positive inhabitants.

*“The one with full protection appears like total war, like in one in operating theatre, whilst this woman in T-shirt and chitenge material appears like a woman dressed to carry out work in the compound, these dressing [full PPE] would make people to know that COVID-19 is there*” (Lusaka, Urban Community Leader).

The diagnosis assumption associated with full PPE created fear for their own health in addition to social stigma. One community leader in Southern Province admitted,

“I know [I] am a civic leader but I would be somehow suspicious because I can see double masks. The first one is covering the mouth, the second one is covering the forehead, thirdly I can see some kind of a gown. So somehow, I would [be] suspicious saying that maybe I have [been] diagnosed with something, have you come to attend to me?”

Equally important to the FGD participants was the concern that full PPE hid the wearer’s identity. The masking of identity with full PPE was proposed as a means for thieves and gassers to enter the community:

*“In many instances when someone comes to you while their face is fully covered, people get to think they are a criminal. They have come with their face hidden, they are a criminal or a crook, why are they hiding, those are people’s thoughts”* (Lusaka, Rural Community Leader).

ZAMPHIA enumerators are not from the community in which they collect data. By further distancing the enumerators from the community by wearing full PPE, FGD participants were less likely to welcome the enumerators into the community as described by one young adult in Southern Province:

“*Honestly, if you don’t know the person like me, the way they are dressed there, he just comes to collect samples from me, I can be scared because see, [I] am not expecting that person, and especially when he comes with a person I don’t know, so it can be hard...to do the services he wants because I do not know you*.”

### Theme 2: Sensitization as a community endorsed method to reduce perceived outsider threat

Across FGDs, participants shared a nearly identical set of recommendations that could be applied to improve community engagement in studies such as ZAMPHIA. They emphasized the importance of increasing community knowledge and understanding of the study through sensitization before any data collection commences.Often, without prompt, FGD participants provided recommendations on mechanisms to build trust, awareness, and acceptance of ZAMPHIA prior to survey administration. For example, when discussing PPE acceptability, participants not only explained why the face covering was acceptable and the full PPE was not, but would continue explaining how the ZAMPHIA team could make the full PPE more acceptable to communities. Regardless of the topic area—enumerators wearing PPE, blood collection and HIV testing, strangers entering the compound—participants suggested community members would be more welcoming and accepting if the underlying questions and unknowns were removed ahead of data collection. They proposed education through sensitization should be centered on two main concepts: providing the detailed information about ZAMPHIA administration, aims, procedures, and timelines in advance; and introducing the ZAMPHIA representatives to community members through relationship building with trusted members of the community, such as compound and church leaders.

In wanting to know more about ZAMPHIA administration, FGD participants described the need for increased transparency surrounding ZAMPHIA procedures. Particularly, participants wanted to know what to expect before the enumerators arrived.

*“Taking part in this survey, I would, or we would accept but there is need for people to know. When people know they can take part, when people do not know that is what would make the people not participate. Participating in this survey can be agreeable but before the survey begins people must be sensitized so that they know why we are doing this; when they know why this [is being] done, they would participate”* (Southern Province, Urban Community Leader).

Participants spoke of the feeling of not knowing and how that leads to unwillingness to accept the ZAMPHIA enumerators or participate in the study. Several FGD participants said participating in the FGD had been the first opportunity to be fully educated about ZAMPHIA, but that participating in the FGD also enabled them to be a spokesperson for the study in the future. One Community Leader in rural Lusaka summed up the group’s concern when discussing ZAMPHIA enumerators arriving in the community:

*“We do not know these people, they are not from here and there was no meeting held to sensitize people about such a program, when it will take place, what will happen is this, then these blood samples will be taken to such a place. That is all I heard, I did not get full information*.”

Participants also wanted to know the results of the HIV blood tests. They expect to get their HIV test results and recommended healthcare follow-up soon after the survey is administered. They recommended mechanisms (see Table 3) to provide robust details across each step of the data and blood sample collection process including: the purpose of the survey and how it may benefit the community; the full data collection process from when an enumerator knocks on the door until the time they leave; how enumerators will be dressed and what identification participants can expect to see; where the blood samples were be taken, what tests will be run, and when participants can expect to be informed of results.

**Table 3:** Consolidated Sensitization Recommendations

**Desired education**	**Participant recommendations**
Purpose of study	Use social media, TV, and radio not just to advertise but to provide details; Leave flyers or pamphlets with the local leadership/gatekeepers; Hold community meetings in which community members can ask questions; Describe the benefit to the individual and the community. *Like I had earlier indicated to say I had inquired after seeing vehicles moving around the community to say what were they doing? Information was not full however, after participating in this focus group discussion today, I now have full information from you, I have known* ~Lusaka, Rural Community Leader
Data collection processes	Schedule all ZAMPHIA visits to the community through the local leadership/gatekeepers; Have a trusted community representative escort enumerators throughout the compound; Provide incentives (e.g. food items, money); Leverage young adult group members in the community to act as role models to increase adolescent participation. *Like when you reach people’s homes, don’t just reach and start doing your programs, first when you reach you first introduce yourselves you say that we are so coming from ZAMPHIA we have to do such and such so that those people should not think that we are also gassing*. ~Southern Province, Peri-Urban Young Adults
Blood collection amounts and process	Describe the amount needed for tests to be accurate; Provide a timeline for test results and when to expect follow-up; Equate the amount being collected to the amount a hospital would collect for the same tests. *People would ask about the amount of blood that is being collected, why is it being collected this way and not like finger pricking like the others had said which requires a little blood*. ~Southern Province, Urban Head of Household
Enumerator dress	Post images of ZAMPHIA enumerators in full PPE at the clinic and community gathering places; Require all enumerators to have a ZAMPHIA ID visible while in the community; Explain the purpose for more PPE than a face covering. *Number one, an identifiable person so that I can trace them, where are they coming from, who are they, that would make me very comfortable. Traceable, I can trace them to some government office, they are not imposters…[there] are many imposters that go round so that will make it a lot easier because you can refer back*. ~Southern Province, Urban Community Leader

The second focus of sensitization participants recommended was through the relationship with trusted community members. Participants recommended increased sensitization through community leaders and institutions. They emphasized the entity communicating ZAMPHIA information was as important as the information itself to community acceptance. As outsiders to the community, ZAMPHIA representatives attempting to educate the community without endorsement of the community leaders would result in further othering of ZAMPHIA enumerators, “*To start with, we can’t believe them because they do not appear to be known so we can’t feel safe*” (Lusaka, Rural Community Leader). Young adult participants recommended use of young adult groups in the community to increase sensitization. Most young adult participants in Southern Province believed strongly that ZAMPHIA is a good cause but more outreach to their age group was needed. They wanted to be a part of the ZAMPHIA process because they wanted to be a model or an example to those younger than them. They believed that this next generation held limited knowledge related to both HIV health and ZAMPHIA.

FGD participants outlined several trusted entities to support sensitization, with the most common being churches, schools, and Headmen or village leaders. They suggested ZAMPHIA be approached as any national initiative, with active advertising and involvement of community and civic leaders such that the local leadership takes on the responsibility for scheduling and information dissemination. Several volunteered themselves as experienced community mobilisers. FGD participants were eager to support ZAMPHIA’s mission, provided increased community involvement:

*“May I take this opportunity of appealing to you and the powers that be…Please involve the local community leaders as well to help in the sensitization, provided you teach us and get to all everything about it. Include all [in] the hierarchy from top to [bottom] for this program to be success[ful]”* (Southern Province, Rural Community Leader).

## Discussion

Study findings illustrate crises (e.g. gassing and COVID-19) enhance mistrust of medical researchers, specifically mistrust surrounding the collection and storage of blood, already woven throughout the fabric of the community. The issue predates the ZAMPHIA study. Rumors of Satanism, selling blood for money, and ritual use of blood are pervasive across many parts of sub-Saharan Africa and represent tensions inherent in medical research throughout the region. Many of these concerns are rooted in beliefs about the power of blood, as necessary for life and its reproduction, and by extension, the potential for its misuse as a means to harm or control others.^13^ These narratives and rumors about blood are understood within communities as representations of “fundamental truths,”^8^ particularly those related to medical research and foreign researchers.^8,14^ Community responses to these concerns should also be viewed within the context of historical oppression.^4^ Therefore, ‘complex accusations’ associated with these beliefs may speak to underlying struggles of power and position and perception of marginalisation.^8^ Further, community actions that result from “rumors,” such as refusal to participate, could be viewed as a form of protest to existing power structures that leave many communities on the periphery.^8^ Dismissing the rumors as mere barriers to research that can be dispelled through knowledge prevents researchers from effectively engaging the community. Rather, taking cultural beliefs seriously and incorporating historical experiences into early sensitization efforts has the power to enhance trust.^15,16^

Recommendations from participants for a comprehensive and multipronged approach to sensitization highlight the importance of having a relationship with the community and understanding the local environment when implementing health interventions and collecting data for medical research. FGD participants suggested meeting with the community before attempting to collect data, organizing visits through headmen, leveraging trusted community members for introductions of enumerators to community members, and providing multiple sources of information prior to beginning the study. These preferences mirror suggestions from other studies that demonstrate early and continuous engagement with the aim to understand the culture and build relationships increases community trust as well as study retention.^17-19^ Arguably, understanding the culture and the community is paramount to any future engagement – it is impossible to effectively engage a community you either do not understand or respect. From this understanding, communication and sensitization strategies can be designed for increased acceptability and reception by a wide range of research stakeholders.^20^ In an example of community engagement to inform processes and protocol, researchers in Luangwa, Zambia offered Community Advisory Board members a tour of the blood receipt and storage facilities and processes to help alleviate concerns related to blood collection ahead of a randomized HIV combination prevention intervention.^21^

Similar to our findings, a study by Zulu and colleagues^22^ found pervasive rumors of Satanism, using untrusted communication channels, and confusion about the study aims all challenged community engagement and led to low survey participation in a school-based pregnancy intervention in rural Zambia. The authors emphasized local leaders are “gate keepers” and important in local communication processes about community activities.^22^ The failure to provide information about the study to community leaders and stakeholders challenged norms and values regarding hierarchy in Zambia and laid grounds for misunderstandings and misinterpretations of information.^22^ Additionally, studies addressing challenges with adolescent recruitment and data collection in HIV studies found social stigmatization, consent from parents, limited knowledge, and fear of rejection by families and friends to contribute to low participation rates among young people.^22-24^ Young adults in our FGDs wanted to act as an example or role model for younger adolescents in the community who were perceived to have both limited health and ZAMPHIA knowledge. Thus, to improve recruitment and reach, it may be important to work across generations when making efforts to engage the community to ensure young community members feel more directly involved and could serve as gate-keepers for a younger participant population.

The role of environmental conditions in the acceptance of study enumerators may have been overemphasized given the selection criteria included regions that had experienced gassing. However, working in these contexts helped to illustrate how crises serve not to alter, but to amplify underlying community concerns that should always be taken seriously and addressed when working at the community level. Using FGDs as part of formative implementation research for future studies may help provide deeper understanding of communities and their priorities and reservations. It is also worth noting that the team that did the analysis was not engaged with the Zambian communities in which FGDs were conducted. This was mitigated through regular coordination and consultation with the field work team throughout the process.

## Conclusion

Lessons for improving community-engaged research abound; however, due to funding, planning constraints, and time limitations, the lessons are rarely fully implemented. This study emphasizes the importance of engaging the community early in study development, and consistently through dissemination of results; not just as a best practice, but as a mechanism to guard against unpredictable environmental influences – gassing incidents and COVID-19 in the case of ZAMPHIA. This study further underscores the actionable recommendation to engage communities early that should be considered for future studies to increase study acceptance and participation rate, particularly in those studies collecting biomarker data.

The findings from this study lend themselves to a number of actionable recommendations. First, communities should be engaged early, and with the intention of relationship building and understanding. Second, effective community engagement requires resources. Our findings underline the importance of prioritizing community engagement through substantial investment in varied and extensive approaches to sensitization to facilitate community engagement toward community acceptance of ZAMPHIA. This study supports the suggestion that we must prioritize, in budgets, the significant investment of time and personnel necessary to do the work of building and maintaining community engagement and trust.^17^ Further, these findings may be used to implement recommended sensitization methods and assess changes in ZAMPHIA acceptance, or used to support rapid assessments to determine and refine the most effective ways of mobilizing the different communities recruited into the study.

## Authors’ Contributions

SHO, EJR, KS analyzed data, developed theme map, drafted manuscript, and approved final version. TS, MC supported data collection teams in Zambia, reviewed analysis to validate representation of participants, drafted sections of the manuscript, and approved final version. CA, KAS, KD, MC supported research design, drafted sections of the manuscript, and approved final version.

## Ethical approval

The study was submitted to and approved by the University of Zambia Biomedical Research Ethics Committee (UNZABREC), National Health Research Authority (NHRA), and the University of Maryland, Baltimore’s (UMB) Institutional Review Board (IRB). All procedures performed in studies involving human participants were in accordance with the 1964 Helsinki declaration and its later amendments.

## Competing Interests

The authors declare that they have no conflict of interest.
